# Typhoid Fever*Read before The Lippe Society.

**Published:** 1888-12

**Authors:** George H. Clark

**Affiliations:** Germantown, Philadelphia


### TYPHOID FEVER.*

GEORGE H. CLARK, M. D., GERMANTOWN, PHILADELPHIA.

A few weeks ago a member of this Society asked: "What are the indicating symptoms in typhoid fever? Which are the symptoms that lead to the remedy?" This may be briefly answered by saying, that, as in all other affections, the peculiar characteristic and striking symptoms, particularly the mental, are the ones to be given most attention.

* Read before The Lippe Society.

The pathological changes in typhoid fever have received so much attention that one is obliged, in treating this affection, in order to fully appreciate the course of the attack, and to be able to judge more knowingly of its progress, to have constantly in view what is, ordinarily, to be anticipated—so far as one can anticipate the various and often unlooked-for changes in disease.

The temperature and pulse are of more moment than even the intestinal lesion, for it will be found that the conditions of these important factors keep equal pace with the lesions in the intestines and the other organs involved.

Hence, it is of primary importance to note and to keep a careful record of both pulse and temperature. It has been asserted by Wunderlich that, to have a genuine case of typhoid fever, there must be a temperature of 103° or 104°. Even though all the symptoms may simulate the condition usually called typhoid fever, without the high temperature it is not that malady, although the character of no disease is better understood than this, almost none having had more attention given to its pathological changes—and none having had more diverse therapeutics proposed—still, it will not be unprofitable to give some little time to its various manifestations, for then we shall be better able to judge which of the symptoms demand closest attention. It is impossible to assert positively that we have a case of typhoid fever until after three or four days of observation, and then only when we find the characteristic evening rise and morning fall of temperature.

Equally impossible is the power to state what may be the course of the attack.

By adhering rigidly to Hahnemann's law in the treatment, it is feasible to turn the whole trouble aside in less than one week, in some cases, while in others, a longer period passes before we see convalescence begin.

In a large number of cases we shall find nervous symptoms predominating. This should not be unexpected, for the high temperature indicates the nervous character of the disease.

Now in all nervous affections, we anticipate contradictory symptoms, and we shall not be disappointed here; for in the majority of instances, particularly in the early stages, the symptoms will oftentimes be very contradictory.

If we possessed the ability of determining beforehand that a low form of fever was approaching, we would be better able to appreciate just what our remedies are capable of doing. For, frequently, we do what we fail to credit ourselves with—that is, we turn aside, and cause to disappear, symptoms of disturbance that are unerring forerunners of various grave affections.

Preceding nervous or low fevers are symptoms which, if we recognize their import, will enable us to do much to modify the subsequent condition. Thus, if you see a persistent dry tongue and no thirst, expect typhoid or some other form of nervous fever. And so with a clean tongue, no appetite, and abnormal taste. The condition of the tongue is of much value in this disease. Knowing the nervous character of the disease, and the nerves which supply the tongue and their functions, and comparing the provings of our remedies, we can more fully appreciate the beauties, and the truly scientific nature of homœopathic treatment. And by giving attention to the origin of the various nerves which supply the affected organs, we become possessed of a knowledge of the sort of practical physiology with which we should all be familiar, and which is of more value than any theorizing.

Even slight observation will show many changes in the tongue during an attack of typhoid fever.

At times during the unconscious state, this is one of our chief guides in prescribing.

The nerves which supply the tongue are the lingual, the hypoglossal, and the glosso-pharyngeal.

The hypoglossal is the motor nerve; the lingual supplies the anterior extremity and middle portion both with general sensibility and the power of taste; and the principal office of the glosso-pharyngeal is to impart the sense of taste to the posterior third.

In typhoid fever motion of the tongue, feeling, and taste are more or less altered. We can thus see what an effect this fever has upon the medulla oblongata, for all of these nerves have their origin there.

Beside all this, the tongue is of peculiar importance in its relation to the alimentary canal and its accessory organs. In most cases it affords definite and valuable information as to their condition. For in typhoid fever there are marked lesions in the intestines and their related organs. Hence it requires no argument to see how very important is close attention to the condition of the tongue.

Mental symptoms, of course, should receive our watchfulness. The most trivial mind-symptoms will frequently enable us to find the remedy which fits the entire condition, and for which we may have vainly sought.

Now, given a continued high temperature, as is found here, what then are we to expect? A melting down of tissue, especially muscular fibre.

Physiology teaches that the "consequence of tissue-wasting is that a large quantity of nitrogenized débris is thrown into the blood, and cast out by the kidneys." Knowing these facts, we must be on the watch for disturbances in other organs than those immediately involved. The waste of muscular fibre goes on not only in the voluntary but in the involuntary muscles. Hence we must expect at some period of the attack a weak heart. The more prolonged the high temperature, the more we shall notice this muscular weakness. Its absence is always of favorable omen. If we can see that much prostration is *not* present, notwithstanding many of the symptoms of the case appear grave, we can feel comfortable about the result.

The liver and spleen are usually involved in this affection, and they may be greatly enlarged.

The intestinal lesions may be approximately gauged by the amount of pain in the ileo-cœcal region and by the character of the stools, which latter must always be carefully inspected, either by a competent nurse, or the physician himself. If it be not the custom to use a note-book, it will be found indispensable here. A careful record of all symptoms, including pulse and temperature, are of much value in assisting one to appreciate what he is doing in the treatment. Although it is asserted by those who are really unfit to take the name of physician that the thermometer is of no value, it will readily be found by him who is eager to know the value of all the factors of disease—so far as they can be known—that this is an instrument that cannot be dispensed with, more particularly in typhoid fever. It, of course, does not lead us to a remedy; but it does show the gravity of the condition of the patient.

What is to be said in respect of "taking the case" applies not only to typhoid fever. The homœopathician adopts this method in all states in treating patients. In truth, he cannot treat any case successfully unless he does adhere to Hahnemann's mode of examination. Dr. Hering repeatedly said to those who were fortunate enough to be his students, when speaking of how to get a proper picture of the patient's state, "*Listen, observe, note* (write), *question.*"

Let us make an indelible mental note of those four words.

Then, with note-book and thermometer we may proceed to examine our patient.

Note all that is to be found regarding the state of the mind. Hallucinations, dreams, illusions, and every alteration in the senses and disposition.

All the various causes of aggravation and amelioration of each simple symptom. Rest, motion, position in bed; the side most affected; and longings, and loathing of every material or mental nature. Time of appearance or disappearence of symptoms. In short, every symptom, both objective and subjective, must be noted and considered.

In the large majority of cases the guiding, most peculiar, striking, and characteristic symptoms will be found in the brain, for the brain is usually profoundly disturbed. From this fact, the older physicians gave the name of nervous fever to typhoid. Give heed to the condition of the heart, and do not permit the patient to use the least exertion for any purpose whatever, as death has followed even slight movement, through more work being thrown on the heart than it was capable of bearing.

Enlarged liver and spleen have been spoken of. The ileocœcal region must receive a large share of attention. But it is superfluous to point out here the reason therefor. If one who attempts to treat a case of typhoid fever without having before him the changes in the intestines and symptoms following thereon he had better resign the case to other hands.

After having carefully gone over the entire condition and noted all that is to be seen and heard with Hering's *Analytical Therapeutics* and Dr. Wells' *Treatise on Typhoid Fever*, published as an appendix to Vol. IV of THE HOMŒOPATHIC PHYSICIAN, you will not fail to cure any curable case.

